# [Retracted] β-elemene decreases cell invasion by upregulating E-cadherin expression in MCF-7 human breast cancer cells

**DOI:** 10.3892/or.2025.9039

**Published:** 2025-12-18

**Authors:** Xian Zhang, Yang Zhang, Yinghua Li

Oncol Rep 30: 745–750, 2013; DOI: 10.3892/or.2013.2519

Following the publication of the above paper, it was drawn to the Editor's attention by a concerned reader that the ‘50 µg/ml ELE’ and ‘100 µg/ml ELE’ data panels shown in Fig. 6A were apparently identical, suggesting that the same data panel had been inserted twice into this figure to show the results from differently performed experiments. Moreover, data featured in the microscopic images in Fig. 2 overlapped with images in a paper that had been published by the same research group in 2013 in the journal *PLoS One*, where the treatment represented was different (treatment with TGF-β1). Upon performing an independent analysis of the data in the Editorial Office, it also came to light that one of the same microscopic images was strikingly similar to a data panel featured in Fig. 4 in another article written by different authors at different research institutes that was published subsequently in the journal *Scientific Reports*, albeit the image was featured in colour in that publication, whereas it had appeared in greyscale in the above paper.

Given the nature of the contentious issues repported above, the Editor of *Oncology Reports* has decided that this paper should be retracted from the Journal on account of a lack of confidence in the presented data. The authors were asked for an explanation to account for these concerns, but the Editorial Office did not receive a reply. The Editor apologizes to the readership for any inconvenience caused.

